# Circulating maternal cortisol levels during vaginal delivery and elective cesarean section

**DOI:** 10.1007/s00404-015-3981-x

**Published:** 2015-12-21

**Authors:** Ylva Vladic Stjernholm, Annie Nyberg, Monica Cardell, Charlotte Höybye

**Affiliations:** Department of Women’s and Children’s Health, Karolinska Institute and Karolinska University Hospital, 171 76 Stockholm, Sweden; Medical School, Karolinska Institute, Stockholm, Sweden; Department of Women’s and Children’s Health, Skåne University Hospital, Lund, Sweden; Department of Endocrinology, Metabolism and Diabetology, Karolinska Institute and Karolinska University Hospital, Stockholm, Sweden

**Keywords:** Cesarean section, Cortisol, Delivery, Stress

## Abstract

**Introduction:**

Maternal S-cortisol levels increase throughout pregnancy and peak in the third trimester. Even higher levels are seen during the physical stress of delivery. Since analgesia for women in labor has improved, it is possible that maternal stress during labor is reduced. The aim of this study was to compare maternal S-cortisol during vaginal delivery and elective cesarean section.

**Materials and methods:**

Twenty healthy women with spontaneous vaginal delivery and healthy women (*n* = 20) undergoing elective cesarean section were included in the study. S-cortisol was measured during three stages of spontaneous vaginal delivery (tvd1, tvd2 and tvd3), as well as before and after elective cesarean section (tcs1 and tcs2).

**Results:**

In the vaginal delivery group, mean S-cortisol at tvd1 was 1325 ± 521 nmol/L, at tvd2 1559 ± 591 nmol/L and at tvd3 1368 ± 479 nmol/L. In the cesarean section group, mean S-cortisol at tcs1 was 906 ± 243 nmol/L and at tcs2 831 ± 257 nmol/L. S-cortisol was higher in the vaginal delivery group at the onset of labor as compared to the cesarean section preoperative group (*p* = 0.006). There were also significant differences between S-cortisol levels postpartum as compared to postoperatively (*p* < 0.001).

**Conclusions:**

Maternal S-cortisol was higher during vaginal delivery compared to elective cesarean section, indicating higher stress levels. A reduction in the hydrocortisone dose at childbirth in women with adrenal insufficiency should be considered, particularly in women undergoing an elective cesarean section.

## Introduction

In the pregnant woman, circulating levels of cortisol, corticosteroid-binding globulin (CBG), corticotropin-releasing hormone (CRH) and adrenocorticotropic hormone (ACTH) are increased compared to the non-pregnant state, resulting in an altered sensitivity of the HPA axis, creating physiological hypercortisolism [1]. The placenta is believed to play an important role by secreting CRH and ACTH, leading to increased levels of cortisol, and unlike hypothalamic CRH, placental CRH is not regulated by negative feedback [2–5]. Instead, rising fetal cortisol stimulates further release of CRH from the placenta [6]. CRH levels increase during pregnancy with the highest levels seen in the third trimester. Levels subsequently decrease to non-pregnant levels within a few hours after childbirth [6–8]. ACTH reaches its highest level during labor and rapidly decreases toward non-pregnant values after delivery [9]. In addition to the elevated levels of CRH and ACTH, other mechanisms contribute to the rise in circulating cortisol. Elevated estrogen levels stimulate the liver production of CBG such that less free cortisol is available for negative feedback, which leads to an enhanced cortisol synthesis [2]. Furthermore, the clearance of cortisol is decreased [3].

Labor is assumed to be a physiologically stressful situation. However, reports on maternal S-cortisol levels during delivery are scarce and only a few studies have been published [3, 8, 10, 11]. In a study from 1976, Goldkrand et al. [10] showed that vaginal delivery (VD) was associated with higher circulating cortisol compared to cesarean section (CS). In 1987, Kono et al. [11] found that maternal cortisol levels correlated with parity and duration of labor.

Adrenal insufficiency (AI) during pregnancy is either primary or secondary, with exogenous corticosteroid therapy as by far the most common etiology. Women with AI are monitored closely during pregnancy and receive increased doses of hydrocortisone during pregnancy and delivery. In women who are treated with steroids, the dose is minimized during pregnancy. In connection with labor and cesarean section, most guidelines recommend standardized supplemental doses of hydrocortisone irrespective of the patients daily prescribed dose. However, symptoms of hypercortisolism such as hypertension, anxiety, restlessness and insomnia after such increases in the cortisone substitution are, in our experience common, indicating that we might be overcompensating the supposed deficit.

Since AI during pregnancy is rare, the present study was designed to quantify maternal S-cortisol in healthy women during spontaneous VD and elective CS. Data were then interpreted in the context of hydrocortisone cortisone replacement therapy with special regard to present-day recommendations.

## Materials and methods

This pilot study was approved by the Ethical Board for Medical Sciences in Stockholm, Sweden, DNr 2013/178-31/4. Between September 1, 2014, and February 28, 2015, 1,889 deliveries and 178 elective CS were registered at the Department of Women’s and Children’s Health, Karolinska University Hospital, Solna, Sweden. Women who were eligible for the study were included after oral and written consent. Maternal S-cortisol levels were measured during spontaneous VD and at elective CS.

### Study participants

Inclusion criteria were healthy women with uncomplicated pregnancies. Exclusion criteria were multiple pregnancy, concomitant diseases such as diabetes mellitus or pituitary disease, ongoing steroid therapy, and pregnancy complications such as preeclampsia or gestational diabetes. The VD group included women (*n* = 20) with spontaneous VD at term gestational age. Peripheral venous samples were obtained at three stages of labor: (tvd1) during the first stage of labor at a cervical dilatation of 4–6 cm, (tvd2) during the second stage of labor when the uterine cervix was fully dilated and (tvd3) during the third stage, i.e., within 2 h after delivery of the placenta. The cervical diameter was continuously assessed by a midwife. The CS group included women (*n* = 20) undergoing elective CS before onset of labor. The most common indication for a planned CS was two previous CS or fear of childbirth. Peripheral venous samples were obtained before and after elective CS: preoperatively (tcs1) and within 2 h postoperatively (tcs2).

Analyses of S-cortisol were performed at the Department of Clinical Chemistry, Karolinska University Hospital, using electrochemical luminescence, Modular E, Roche Diagnostics, Mannheim, Germany.

Clinical data regarding maternal age, body mass index (BMI), parity, gestational age, duration of labor, indications for and duration of CS, type of analgesia such as epidural analgesia (EDA), spinal analgesia (SPA), inhalation of nitrous oxide (N_2_O) in combination with oxygen or other medication were obtained from computerized medical records.

### Statistics

Data were presented as mean and standard deviation (SD). For comparison between groups, Student’s *t* test was used for normally distributed data and Mann–Whitney *U* test for non-normally distributed data. *p* values of <0.05 were considered significant.

## Results

Clinical data are shown in Table 1. The maternal age and proportion of primiparity did not differ, whereas the mothers in the CS group had a higher BMI (*p* = 0.005). The duration of VD ranged between 185 and 1075 min, mean 624 min. The average duration in primiparous women (*n* = 13) was 728 min and in parous women with one previous vaginal delivery (*n* = 6) 394 min. One parous woman with a medical history of one previous VD and one CS had a delivery lasting 661 min. The duration of the elective CS ranged between 17 and 60 min with an average of 37 min.

**Table 1:** Clinical data

Variable	VD (*n* = 20)	CS (*n* = 20)	*p* value
Maternal age (years) (median and range)	33 (23–42)	33 (24–43)	>0.05
BMI (kg/m^2^) (median and range)	23 (18–28)	26 (21–34)	>0.05
Primiparous (%)	13 (65)	7 (35)	>0.05
Gestational age (weeks) (median and range)	40 (38–41)	39 (37–39)	>0.05
Regional anesthesia (%)	15 (75)	20 (100)	>0.05
Birth weight (g) (mean ± SD)	3617 ± 457	3492 ± 452	>0.05
Apgar score <7 at 5 min	0	0	>0.05

**Fig. 1:**
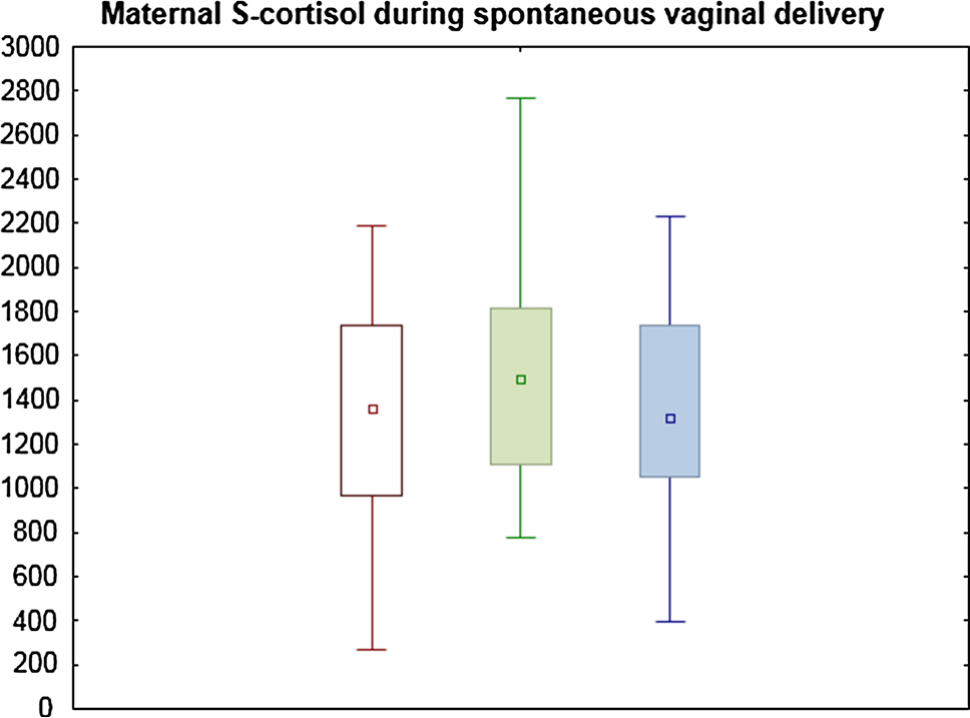
Maternal S-cortisol during spontaneous vaginal delivery

**Fig. 2:**
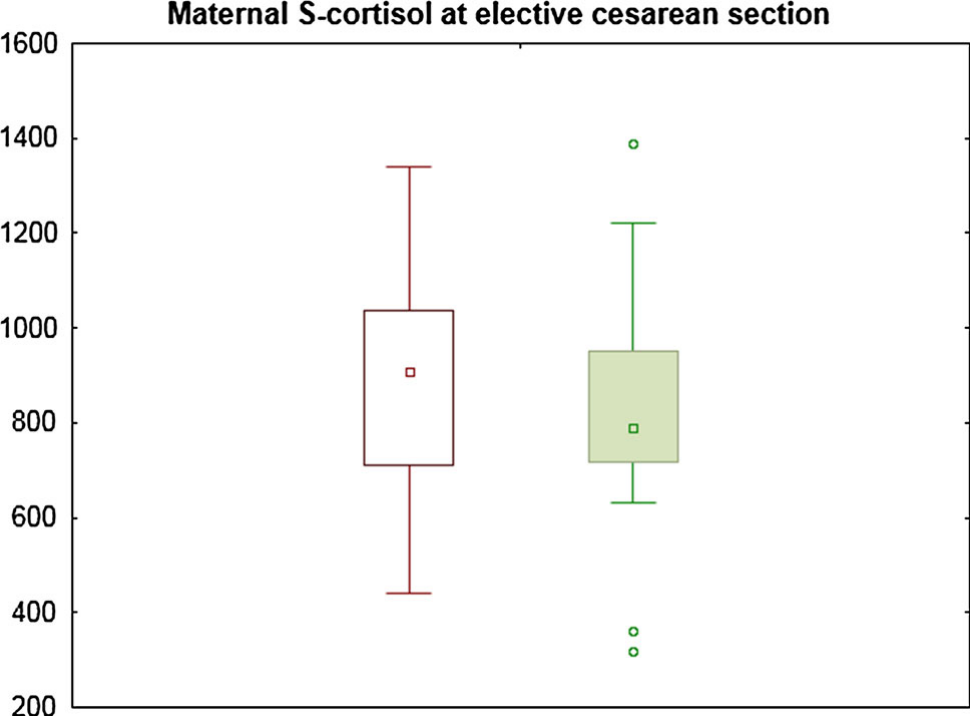
Maternal S-cortisol at elective cesarean section

Maternal S-cortisol levels are shown in Table 2, Figs. 1 and 2. In the VD group, mean S-cortisol at tvd1 was 1325 ± 521 nmol/L, at tvd2 1559 ± 591 nmol/L and at tvd3 1368 ± 479 nmol/L (Fig. 1). There were no significant differences between the three measurements. There was no correlation between S-cortisol at tvd1, tvd2 or tvd3 and maternal age, BMI, parity, gestational age or duration of labor (data not shown). The highest S-cortisol level was 2710 nmol/L, observed during the second stage of labor (tvd2). A peak S-cortisol was observed at tvd1 in 6 women (30 %), at tvd2 in 10 women (50 %) and at tvd3 in 4 women (20 %). The largest increment was 44 %, from 1200 nmol/L at tvd1 to 2710 nmol/L at tvd2. Notably, S-cortisol was decreased during the second stage of labor in six women.

**Table 2:** Maternal S-cortisol (nmol/L, mean ± SD)

	tvd1	tvd2	tvd3	tcs1	tcs2
VD	1325 ± 521*	1559 ± 591	1368 ± 479**		
CS				906 ± 243*	831 ± 257**

Sample tvd1 during the first stage of labor, tvd2 during the second stage and tvd3 during the third stage. Sample tcs1 before elective CS, tcs2 within 2 h, postoperatively

* *p* = 0.06; ** *p* < 0.001

In the CS group, mean S-cortisol was 906 ± 243 nmol/L at tcs1 and 831 ± 257 nmol/L at tcs 2 (Fig. 2). There were no significant differences between these measurements (*p* = 0.352). There was no correlation between S-cortisol at tcs1 or tcs2 and maternal age, BMI, parity, gestational age or duration of surgery (data not shown). A peak S-cortisol level was observed at tcs1 in 60 % and at tcs2 in 40 %. The highest value was 1390 nmol/L, observed postoperatively (tvd2). The difference between maternal S-cortisol at tvd1 in the VD group and tcs1 in the CS group was significant (*p* = 0.006) as well as the difference between tvd3 in the VD group and tcs2 in the CS group (*p* < 0.001).

All women undergoing VD were offered labor analgesia including acupuncture, inhalation of N_2_O 50 % in combination with oxygen 50 % and epidural anesthesia (EDA). Fifteen women (75 %) in the VD group received EDA. Five women received only acupuncture and/or N_2_O with oxygen. According to the medical records, 13 women (65 %) in the VD group received EDA during the first stage of labor. There was no correlation between S-cortisol and different modes of analgesia (data not shown). Two women were delivered by ventouse due to a prolonged second stage of labor. In these women, S-cortisol levels were comparable to the levels during unassisted delivery. In the elective CS group, all women received spinal anesthesia (SPA) before surgery.

## Discussion

In this study, we monitored serial maternal S-cortisol in healthy women with uncomplicated pregnancies throughout the different stages of VD, as compared to elective CS before the onset of labor. S-cortisol during VD was comparable to the values reported in previous studies, whereas the levels at elective CS were lower than was reported previously. However, only single measurements were taken in earlier studies. We found that spontaneous VD generated higher maternal S-cortisol than elective CS, indicating higher stress levels. We found no correlation between S-cortisol and maternal age, BMI, parity, gestational age or duration of labor.

A two- to fourfold increase in S-cortisol from 336 ± 16 nmol/L at 16 gestational weeks (*n* = 33) to 810 ± 82 nmol/L at 38 gestational weeks (*n* = 31) has been reported [12]. A variation in S-cortisol between 450 and 1518 nmol/L in the third trimester in normal pregnancies has been described [13]. The S-cortisol levels before elective CS in the present study tcs1 were comparable to the third trimester levels, as reported by Ho et al. [12]. There are no previous reports on S-cortisol levels at emergency CS. However, S-cortisol levels during emergency CS performed before active labor would be similar to the levels observed during the first stage in the VD group (tvd1). Likewise, S-cortisol levels at emergency CS carried out during the active labor would be similar to the values observed during the second stage of labor in the VD group (tvd2).

In 1976, Goldkrand et al. [10] showed that maternal S-cortisol levels in 95 women were higher after VD as compared to after CS. They found that S-cortisol measured immediately after uncomplicated VD averaged at 52.2 μg/100 mL (1440 nmol/L). In the CS group, mean S-cortisol values were 48.3 μg/100 mL (1333 nmol/L). In 1987, Kono et al. showed that maternal S-cortisol levels in 130 women during VD were higher in primiparous women (64.3 µg/100 L (=1774.0 nmol/L) than in parous women (37.8 µg/100 mL (=1042.9 nmol/L) [11]. We did not find such a correlation, which might be due to the smaller sample size in our study. We hypothesized that the introduction of labor analgesia during the last decades might result in a reduction in maternal stress during delivery [14]. If so, lower S-cortisol levels at VD would be expected in today’s clinical practice. S-cortisol values in the VD group were, however, comparable to the levels reported in previous studies. S-cortisol levels in the CS group were lower at tcs 1 and tcs2 as compared to the VD group and compared to previous studies. These findings indicate that VD is more stressful than elective CS.

According to international guidelines, women with AI at the Karolinska University Hospital receive hydrocortisone 100–300 mg throughout labor and 24 h after delivery [2, 15]. After delivery, there is a reduction in the prepregnancy treatment dose over 48 h [2, 15–18]. The indications for CS in women with AI are similar to those in healthy women [2, 15–18]. Björnsdottir et al. [19] reported an increased risk of premature delivery and a higher frequency of CS in women with Addison’s disease.

The reports on S-cortisol following intravenous (i v) injection of hydrocortisone are limited. In a study conducted in men, S-cortisol measured 10 min after an i v injection of hydrocortisone, 20 mg, was 1059 ng/mL (2921.7 nmol/L), whereas S-cortisol was 1854 ng/mL (5115 nmol/L) after an i v injection of 40 mg hydrocortisone [20]. Our results, in combination with previous reports and taking into account complaints regarding symptoms of hypercortisolism, suggest that the recommended hydrocortisone doses at childbirth for women with AI may be too high. Therefore, a reduction in the hydrocortisone dose at childbirth in women with AI should be considered, particularly in the case of an elective CS. However, the optimal hydrocortisone dose has yet to be decided and should be adjusted to individual requirements. In this study, S-cortisol levels were not quantified during term pregnancy or during emergency CS. However, the S-cortisol levels observed before elective CS are comparable to the levels observed during term pregnancy, as shown by Ho et al. [12].

The strengths of this study were the consecutive S-cortisol monitoring during VD and at elective CS. To our knowledge, this is the first report on consecutive sampling of maternal S-cortisol. Due to practical reasons, samples were obtained at relatively long intervals. A more frequent sampling regime would probably not have changed the results significantly.

The small sample size was a limitation. Therefore, correlations might have been detected between S-cortisol and different types of analgesia, maternal age, BMI, parity or duration of labor in a larger cohort. As in previous studies, total S-cortisol was measured and individual differences in the ratio between free (active) and CBG-bound cortisol were not determined. Saliva cortisol sampling may be a way to deal with this issue [20]. A quantification of S-cortisol after an i v injection, or after oral administration of a double dose of hydrocortisone in women with cortisol insufficiency, would be valuable in deciding on an optimal dose regime in connection with childbirth.

In conclusion, S-cortisol levels were significantly higher in women who underwent spontaneous VD compared to elective CS before the onset of labor, indicating that VD is more stressful than an elective CS. A reduction in the hydrocortisone dose administered at childbirth in women with AI should be considered, particularly in the case of elective CS. Larger studies exploring the S-cortisol levels in women with different parity, as well as in women undergoing emergency CS after the onset of labor might be helpful in establishing more precise guidelines for hydrocortisone treatment at childbirth in women with AI.

## Abbreviations

AI: Adrenal insufficiency
ACTH: Adrenocorticotropic hormone
CS: Cesarean section
CBG: Corticosteroid-binding globulin
CRH: Corticotropin-releasing hormone
EDA: Epidural analgesia
SPA: Spinal analgesia
VD: Vaginal delivery
